# Correction: Molecular simulation-based investigation of thiazole derivatives as potential LasR inhibitors of *Pseudomonas aeruginosa*

**DOI:** 10.1371/journal.pone.0330995

**Published:** 2025-08-25

**Authors:** Snigdha Bhardwaj, Kandasamy Nagarajan, Halima Mustafa Elagib, Sadaf Anwar, Mohammad Zeeshan Najm, Tulika Bhardwaj, Mohd Adnan Kausar

The affiliation for the second author is incorrect. Kandasamy Nagarajan is not affiliated with #2 but with #1: KIET School of Pharmacy, KIET Group of Institutions, Ghaziabad, Uttar Pradesh, India.
