# Supplementary material for: Molecular simulation-based investigation of thiazole derivatives as potential LasR inhibitors of Pseudomonas aeruginosa
Source: PLoS One. 2025 Apr 22;20(4):e0320841. doi: 10.1371/journal.pone.0320841 (PMC12061423; doi:10.1371/journal.pone.0320841)
Supplement: Attachment — Submitted filename: Response to Reviewers.docx [file pone.0320841.s003.docx]

Response to Reviewer

**A. Introduction and Background**:

***1. While the introduction is well-rounded, it could further emphasize the novelty of focusing exclusively on thiazole derivatives over other compound classes previously reported for quorum sensing inhibition.***

**Response.** The detailed explanation has been added on page number 7 (Red coloured text)

***2. Expand on how this work compares or improves upon prior studies in the field.***

**Response.** The detailed explanation has been added on page number 8 (Red coloured text)

**B. Molecular Dynamics Details**:
***- The manuscript discusses 50 ns molecular dynamics simulations but does not clearly justify why this timespan was chosen or if longer simulations were considered.
- Further discussion on the stability and interactions observed during simulations could be beneficial****.*

**Response.** Authors are thankful to the reviewer for valuable comment. The 50 ns MD simulation was selected for its ability to capture the conformational stability, dynamic behaviour, and major interactions between the protein and ligand accurately. Simulation lengths of this duration have already been shown in similar studies [1,2] to have been sufficient for the analysis of RMSD, RMSF, hydrogen bonding, and other interaction metrics. Furthermore, 10 ns intervals were selected for the temperature-control simulations to guarantee a good balance between speed and accuracy. The results showed stable trajectories with minor fluctuations in the RMSDs, indicating that the system had reached thermal equilibrium at 300 K. The analyses of hydrogen bonds and protein-ligand contacts showed consistent stability and binding interactions even within this timeframe. Although further simulated trajectories would provide sufficient insights into more infrequent conformational changes or dynamic events, the present findings based on the 50 ns simulation approached the objectives of this study. Future work may involve extended simulations to promote the observation of relatively complex or long-term molecular events.

Furthermore, the discussions regarding the stability and interaction of the system (RMSD, RMSF, hydrogen bonds, and protein-ligand contacts) are already included in detail and supporting scientific explanations at Section 3.5 of Results and Discussion.

References

1.Wang X, Chen Y, Zhang S, Deng JN (2022) Molecular dynamics simulations reveal the selectivity mechanism of structurally similar agonists to TLR7 and TLR8. PLoS ONE 17(4): e0260565. <https://doi.org/10.1371/journal.pone.0260565>
2.Ghahremanian S, Rashidi MM, Raeisi K, Toghraie D. Molecular dynamics simulation approach for discovering potential inhibitors against SARS-CoV-2: A structural review. J Mol Liq. 2022 May 15;354:118901. doi: 10.1016/j.molliq.2022.118901. Epub 2022 Mar 9. PMID: 35309259; PMCID: PMC8916543.

***C. ADMET Analysis:***

***- While pharmacokinetic and toxicity parameters were considered, it is unclear if experimental validation (e.g., stability in biological fluids) will be pursued in future work.***

**Response.** Authors are thankful for insightful comments. Although the study primarily relies on the *in-silico* based pharmacokinetic model building and the toxicity evaluation part, experimental validation will serve a crucial role to gain a translational impact on the study. Our future research experimentation will be focussed on analysing screened compounds stability in biologically relevant fluids (e.g., plasma, gastric fluid) using recommended experimental approaches to infer insights of crucial importance on the relevance and robustness of the findings, which would further boost the translational capacity of the same.

Accordingly, we have made justified additions in the manuscript (Conclusion) highlighting this valuable suggestion.

***D. Conclusions:***

***The conclusions could benefit from a stronger emphasis on the implications of this work and a discussion of the next steps, such as experimental validation or broader applications.
Consequently, the study is primarily computational. The study should acknowledge the inherent limitations of in silico approaches, including the reliance on computational predictions that may not fully replicate biological complexities, and emphasizes the need for experimental validation to confirm the efficacy and safety of the identified compounds in real-world biological systems. Authors should expand on molecular dynamics findings and include additional validation metrics if available (e.g., simulation run length should be 100 ns or Molecular Dynamics (MD) Simulations should be performed on 5 compounds). A revised manuscript should diligently comprehensively address the aforementioned concerns.***

**Response.** Authors strongly agree with reviewer’s comment. Accordingly, justified additions are made in the conclusion section of the manuscript (highlighted in red).
