# Supplementary material for: Molecular simulation-based investigation of thiazole derivatives as potential LasR inhibitors of Pseudomonas aeruginosa
Source: PLoS One. 2025 Apr 22;20(4):e0320841. doi: 10.1371/journal.pone.0320841 (PMC12061423; doi:10.1371/journal.pone.0320841)
Supplement: Attachment — Submitted filename: FINAL_Commenst to reviewer_PLOSone.docx [file pone.0320841.s002.docx]

**Review Comments to the Author**

Please use the space provided to explain your answers to the questions above. You may also include additional comments for the author, including concerns about dual publication, research ethics, or publication ethics. (Please upload your review as an attachment if it exceeds 20,000 characters)

Reviewer #1: Snigdha Bhardwaj and colleagues reported an in-silico screening of possible inhibitors of the Quorum Sensing system in P. aeruginosa. The study described different approaches to eventually identify 29 compounds that were better investigated for their pharmacokinetics and toxicity in silico.

**Actually, the study focuses on thiazole derivatives and LasR interaction. Therefore, the Authors need to better clarify in their study why they decided to take into consideration only thiazole derivatives (actually, several different compounds have been reported in QS inhibition in P. aeruginosa) and why they focused on LasR system. Given the receptor specificity of the study, I suggest introducing “LasR” in the title of the Manuscript (something like “thiazole derivatives as potential LasR inhibitors of Pseudomonas aeruginosa”)**

Title has been changed to “Molecular simulation-based investigation of thiazole derivatives as potential LasR inhibitors of *Pseudomonas aeruginosa*”

**The study is rich in different approaches; however, the Authors did not mention about the limitations of the study and did not describe the methods used in data interpretation. Moreover, as the results were obtained in silico, the meanings of the study need to be re-considered, and the last sentence (lines 457-459) needs to be carefully reconsidered.**

The last statement has been revised with clear explanation (Page no. 26, red coloured text).

**Several others major points need attention:**

1. **In the Introduction (line 73), the Authors wrote that P. aeruginosa possesses two unique QS circuits. Actually, four QS pathways were identified in P. aeruginosa, namely Las, Rhl, Pqs, and Iqs (DOI: 10.1126/science.aaw1629).Moreover, in P. aeruginosa, QS controls not only biofilm formation but also and rhamnolipids, pyocyanin, and pyoveridin production. Authors should upload the information.**

The detail for QS circuit/pathways that P. aeruginosa possesses (reference 11,12- on page 4-5) has been added along with the details of different virulent factors (reference 18,19) on page 5-6.

**References**:

Miranda SW, Asfahl KL, Dandekar AA, Greenberg EP. Pseudomonas aeruginosa Quorum Sensing. Adv Exp Med Biol. 2022;1386:95-115. doi: 10.1007/978-3-031-08491-1_4. PMID: 36258070; PMCID: PMC9942581.

Welsh MA, Blackwell HE. Chemical Genetics Reveals Environment-Specific Roles for Quorum Sensing Circuits in Pseudomonas aeruginosa. Cell Chem Biol. 2016 Mar 17;23(3):361-9. doi: 10.1016/j.chembiol.2016.01.006. Epub 2016 Feb 18. PMID: 26905657; PMCID: PMC4798878.

Moura-Alves P, Puyskens A, Stinn A, Klemm M, Guhlich-Bornhof U, Dorhoi A, Furkert J, Kreuchwig A, Protze J, Lozza L, Pei G, Saikali P, Perdomo C, Mollenkopf HJ, Hurwitz R, Kirschhoefer F, Brenner-Weiss G, Weiner J 3rd, Oschkinat H, Kolbe M, Krause G, Kaufmann SHE. Host monitoring of quorum sensing during *Pseudomonas aeruginosa* infection. Science. 2019 Dec 20;366(6472):eaaw1629. doi: 10.1126/science.aaw1629. PMID: 31857448.

Raman Pachaiappan, Tharun Prasanna Rajamuthu, Ananya Sarkar, Pradiksha Natrajan, Nagasathiya Krishnan, Meenakumari Sakthivelu, Palaniyandi Velusamy, Palaniappan Ramasamy, Subash C.B. Gopinath, N-acyl-homoserine lactone mediated virulence factor(s) of Pseudomonas aeruginosa inhibited by flavonoids and isoflavonoids, Process Biochemistry, Volume 116, 2022, Pages 84-93, ISSN 1359-5113, https://doi.org/10.1016/j.procbio.2022.02.024.

2. **The Authors should better describe the advantages of studying the docking in both CviR and LasR: what do these data mean for the present study as they gave different docking scores even against the TC?**

The advantages of studying the docking in both CviR and LasR has been added under point 3.1 (reference 68) on page 12-13.

**Reference**

Baloyi IT, Adeosun IJ, Yusuf AA, Cosa S. In Silico and In Vitro Screening of Antipathogenic Properties of Melianthus comosus (Vahl) against Pseudomonas aeruginosa. Antibiotics (Basel). 2021 Jun 5;10(6):679. doi: 10.3390/antibiotics10060679. PMID: 34198845; PMCID: PMC8230066.

3. **Pharmacokinetic and Toxicity (ADME/T) Studies should also include studies about the stability of the potential inhibitors. Considering the administration route, are the compound stable in biological fluids?**

The description about the stability aspects of the potential inhibitors through ADMET studies have been added under point 3.3 (reference 80,81,82) on page 17.

**References**

George Lambrinidis, Theodosia Vallianatou, Anna Tsantili-Kakoulidou, In vitro, in silico and integrated strategies for the estimation of plasma protein binding. A review, Advanced Drug Delivery Reviews, Volume 86, 2015, Pages 27-45, ISSN 0169-409X, https://doi.org/10.1016/j.addr.2015.03.011.

Nisha CM, Kumar A, Nair P, Gupta N, Silakari C, Tripathi T, Kumar A. Molecular Docking and In Silico ADMET Study Reveals Acylguanidine 7a as a Potential Inhibitor of β-Secretase. Adv Bioinformatics. 2016;2016:9258578. doi: 10.1155/2016/9258578. Epub 2016 Apr 10. PMID: 27190510; PMCID: PMC4842033.

Zakari Ya'u Ibrahim, Adamu Uzairu, Gideon Shallangwa, Stephen Abechi, Molecular docking studies, drug-likeness and in-silico ADMET prediction of some novel β-Amino alcohol grafted 1,4,5-trisubstituted 1,2,3-triazoles derivatives as elevators of p53 protein levels, Scientific African, Volume 10, 2020, e00570, ISSN 2468-2276, https://doi.org/10.1016/j.sciaf.2020.e00570.

4. **Line 300: The authors should detail why they selected the value of -12.0 kcal/mol. Maybe changing the reference compound TC would change the cut-off values, too?**

The justification of selecting cut off value is added under point 3.2 (reference 79) on page 16.

**Reference**

Khidre, R.E., Radini, I.A.M. Design, synthesis and docking studies of novel thiazole derivatives incorporating pyridine moiety and assessment as antimicrobial agents. *Sci Rep* 11, 7846 (2021). <https://doi.org/10.1038/s41598-021-86424-7>

5. **Line 308: The main text does not report Table 1. The Reviewer cannot review the table. Table should report the docking score values for the 800 test compounds (maybe grouped by range values) plus more details (molecular structures) about the 29 selected molecules. Line 324: at the same, Table 2 is not reported in the main text and the Reviewer was not able to review the Table 2. Line 333: the same for Table 3**

All the Tables 1,2,3 provided as a separate document. Please see “other” file.

Secondly, providing Table with the docking score values for all 800 test compounds (even with grouped range values) in the manuscript will not put much impact as we have justified in the manuscript for selecting compounds with cut off value above or equal -12 kcal/mol to extract the potential compounds having good binding affinity with LasR receptor**.** So employing those shortlisted hits in the study.

6. **The results of the Molecular Dynamics Simulations (para 3.5) are not described; it is not clear which molecules are involved in the complex formation. The Authors should justify the parameters set for the experiments (10 ns).**

The justification of protein -ligand complex formation along with parameter details is added under point 3.5 (reference 84) on page 18.

**Reference**

Hollingsworth SA, Dror RO. Molecular Dynamics Simulation for All. Neuron. 2018 Sep 19;99(6):1129-1143. doi: 10.1016/j.neuron.2018.08.011. PMID: 30236283; PMCID: PMC6209097.

7. **Line 372: To improve comprehension, Authors should better describe what they mean as “ligand”.**

Explain in description under point 3.5 (reference 84) on page 18.

8. **No description of the results has been provided for Protein-ligand Contacts (para 3.5.4).**

The description for Protein-ligand Contacts have been added under point 3.5.4 on page 20,21.

**Minor points- Revised as coloured red**

1. **Line 229: name of bacteria in italic**- DONE
2. **Line 230. What is C.?** - DONE
3. **Line 320: ouome?** - DONE
4. **Line 342: closing bracket is missing** - DONE
5. **Line 371: please correct the mistake** - DONE
6. **Line 418: please, better formulate the concept “The chemical D_152's ability to detect quorum” as it is not clear** - DONE
7. **Line 425, 443, and 457: name of bacteria in italic** - DONE
8. **Figures 1, 2, 10, 11, 12 are not mentioned in the main text** - DONE

Reviewer #2: Editorial Office

The manuscript needs minor revision. Detailed explanation of some general and specific points should be given:

Comments

1. **The manuscript needs careful proofreading to correct some punctuation and spelling errors.**

Authors agree with the reviewer’s suggestion and justified modifications are made in the manuscript.

2. **While the manuscript is generally well-organized and clear, breaking down some long sentences could enhance readability.**

Revised wherever applicable all over the manuscript.

3. **Minor corrections are needed in the abstract. Emphasize the aim, novelty, and outcomes of the work.**

Abstract revised with special emphasis on aim, novelty and outcome (Page 2-3).

4. **The introduction requires enhancement. Please provide more insight into the rationale behind selecting the specific compounds for your study.**

The insight into the rationale behind selecting the specific compounds for this study has been added (reference 33-37) on page 7.

**References**

R Santosh, MK Selvam, SU Kanekar, GK Nagaraja, M Kumar. [Design, Synthesis, DNA Binding, and Docking Studies of Thiazoles and Thiazole‐Containing Triazoles as Antibacterials](Design,%20Synthesis,%20DNA%20Binding,%20and%20Docking%20Studies%20of%20Thiazoles%20and%20Thiazole‐Containing%20Triazoles%20as%20Antibacterials) ChemistrySelect 3 (14), 3892-3898. <https://doi.org/10.1002/slct.201800222>

Kartsev V, Geronikaki A, Zubenko A, Petrou A, Ivanov M, Glamočlija J, Sokovic M, Divaeva L, Morkovnik A, Klimenko A. Synthesis and Antimicrobial Activity of New Heteroaryl(aryl) Thiazole Derivatives Molecular Docking Studies. *Antibiotics*. 2022; 11(10):1337. https://doi.org/10.3390/antibiotics11101337

Ratrey P, Das Mahapatra A, Pandit S, Hadianawala M, Majhi S, Mishra A, Datta B. Emergent antibacterial activity of *N*-(thiazol-2-yl)benzenesulfonamides in conjunction with cell-penetrating octaarginine. RSC Adv. 2021 Aug 25;11(46):28581-28592. doi: 10.1039/d1ra03882f. PMID: 35478531; PMCID: PMC9038147.

Abdelsamie AS, Hamed MM, Schütz C, Röhrig T, Kany AM, Schmelz S, Blankenfeldt W, Hirsch AKH, Hartmann RW, Empting M. Discovery and optimization of thiazole-based quorum sensing inhibitors as potent blockers of Pseudomonas aeruginosa pathogenicity. Eur J Med Chem. 2024 Oct 5;276:116685. doi: 10.1016/j.ejmech.2024.116685. Epub 2024 Jul 16. PMID: 39042991.

More, P.G., Karale, N.N., Lawand, A.S. *et al.* Synthesis and anti-biofilm activity of thiazole Schiff bases. *Med Chem Res* **23**, 790–799 (2014). <https://doi.org/10.1007/s00044-013-0672-7>

5. **Highlight the advantages of Molecular docking calculations in the relevant sections, Refer to and cite the following related papers that utilized advantages of Molecular docking calculations to enhance the Molecular docking part:**

The advantages of Molecular docking calculations under introduction part has been added (reference 24-26) on page 6.

**References**

Ahmed M. El-Saghier, Aly Abdou, Mounir A. A. Mohamed, Hany M. Abd El-Lateef, and Asmaa M. Kadry, Novel 2-Acetamido-2-ylidene-4-imidazole Derivatives (El-Saghier Reaction): Green Synthesis, Biological Assessment, and Molecular Docking. *ACS Omega* 2023 *8* (33), 30519-30531 DOI: 10.1021/acsomega.3c03767

Ranjan K. Mohapatra, Ahmed Mahal, Azaj Ansari, Manjeet Kumar, Jyoti Prakash Guru, Ashish K. Sarangi, Aly Abdou, Snehasish Mishra, Mohammed Aljeldah, Bashayer M. AlShehail, Mohammed Alissa, Mohammed Garout, Ahmed Alsayyah, Ahmad A. Alshehri, Ahmed Saif, Abdulaziz Alqahtani, Fahd A. Alshehri, Aref A. Alamri, Ali A. Rabaan, Comparison of the binding energies of approved mpox drugs and phytochemicals through molecular docking, molecular dynamics simulation, and ADMET studies: An in silico approach, Journal of Biosafety and Biosecurity, Volume 5, Issue 3, 2023, Pages 118-132, ISSN 2588-9338, https://doi.org/10.1016/j.jobb.2023.09.001.

Ahmed M. El‐Saghier, Souhaila S. Enaili, Aly Abdou, Amany M. Hamed, Asmaa M. Kadry, An operationally simple, one‐pot, convenient synthesis, and in vitro anti‐inflammatory activity of some new spirotriazolotriazine derivatives, Journal of Heterocyclic Chemistry, Volume 61, Issue 1, 2024, Pages 146-162, ISSN 0022-152X, <https://doi.org/10.1002/jhet.4752>.

6. **It is important to give the reason for why selected the used PDB ID protein in this study**

The justification for the selection of used PDB ID protein in this study has been added under 2.2 heading “Virtual Screening based on Structure Similarity” (reference 54) on page 10.

**Reference**

Bottomley MJ, Muraglia E, Bazzo R, Carfì A. Molecular insights into quorum sensing in the human pathogen Pseudomonas aeruginosa from the structure of the virulence regulator LasR bound to its autoinducer. J Biol Chem. 2007 May 4;282(18):13592-600. doi: 10.1074/jbc.M700556200. Epub 2007 Mar 15. PMID: 17363368.

7. **Clarify the preparation of the protein PDB file for docking. Were additional ligands or water molecules removed? Were hydrogen atoms and charges added? Was the 3D optimized output used for docking the compounds ‘structures? Explain this in the relevant section. Refer to and cite the following papers to enhance the docking part:(**

The process of preparation of the protein PDB file for docking has been added under The below mentioned suggested references are added as 45,46,47 in the manuscript, under 2.1.3. Protein Preparation on pages 8-9.

**References**

El-Saghier, A.M., Enaili, S.S., Kadry, A.M. *et al.* Green synthesis, biological and molecular docking of some novel sulfonamide thiadiazole derivatives as potential insecticidal against *Spodoptera littoralis*. *Sci Rep* **13**, 19142 (2023). <https://doi.org/10.1038/s41598-023-46602-1>

Hany M. Abd El-Lateef, Ali M. Ali, Mai M. Khalaf, Aly Abdou. New iron(III), cobalt(II), nickel(II), copper(II), zinc(II) mixed-ligand complexes: Synthesis, structural, DFT, molecular docking and antimicrobial analysis, Bull. Chem. Soc. Ethiop. 2024, 38(1), 147-166. <https://dx.doi.org/10.4314/bcse.v38i1.12>

[Hany M. Abd El-Lateef](https://onlinelibrary.wiley.com/authored-by/Abd+El%E2%80%90Lateef/Hany+M.), [Mai M. Khalaf](https://onlinelibrary.wiley.com/authored-by/Khalaf/Mai+M.), [Amer A. Amer](https://onlinelibrary.wiley.com/authored-by/Amer/Amer+A.), [Antar A. Abdelhamid](https://onlinelibrary.wiley.com/authored-by/Abdelhamid/Antar+A.), [Aly Abdou](https://onlinelibrary.wiley.com/authored-by/Abdou/Aly). Antibacterial, antifungal, anti-inflammatory evaluation, molecular docking, and density functional theory exploration of 2-(1*H*-benzimidazol-2-yl)guanidine mixed-ligand complexes: Synthesis and characterization. Appl. Organomet. Chem, [Volume38, Issue1](https://onlinelibrary.wiley.com/toc/10990739/2024/38/1) ,January 2024, e7299 <https://doi.org/10.1002/aoc.7299>

8. **Include the IUPAC names for the compounds.**

The IUPAC names are added in the table no. 1 for shortlisted derivatives.

9. **The conclusions are generally well-supported by the data and analyses presented.**

**NA**

10. **Highlight the advantages of DFT calculations and the HOMO-LUMO, Hardness, MEP calculations in the relevant section, and cite the following papers that utilized DFT calculations. Refer to and cite the following articles to enhance the DFT part:**

Advantages of DFT calculations and the HOMO-LUMO, Hardness, MEP calculations in the relevant section has been added as red coloured text. The below mentioned suggested references [as 90,92,93,94 in the manuscript, pages from 21-24] have been added to support the information**.**

**References**

Aly Abdou, Omran A. Omran, Jabir H. Al-Fahemi, Rabab S. Jassas, Munirah M. Al-Rooqi, Essam M. Hussein, Ziad Moussa, Saleh A. Ahmed, Lower rim thiacalixarenes derivatives incorporating multiple coordinating carbonyl groups: Synthesis, characterization, ion-responsive ability and DFT computational analysis, Journal of Molecular Structure, Volume 1293, 2023, 136264, ISSN 0022-2860, <https://doi.org/10.1016/j.molstruc.2023.136264>.

Bendjeddou, A & Abbaz, T & Gouasmia, Abdelkrim & Villemin, Didier. (2016). Molecular Structure, HOMO-LUMO, MEP and Fukui Function Analysis of Some TTF-donor Substituted Molecules Using DFT (B3LYP) Calculations. International Research Journal of Pure and Applied Chemistry. 12. 1-9. 10.9734/IRJPAC/2016/27066.

El-Lateef, Hany & Khalaf, Mai & Heakal, Fakiha & Abdou, Aly. (2023). Fe(III), Ni(II), and Cu(II)-Moxifloxacin-Tri-Substituted Imidazole Mixed Ligand Complexes: Synthesis, Structural, DFT, Biological, and Protein-Binding Analysis. Inorganic Chemistry Communications. 158. 111486. 10.1016/j.inoche.2023.111486.

[Mahmoud Abd El Aleem Ali Ali El-Remaily](https://onlinelibrary.wiley.com/authored-by/El%E2%80%90Remaily/Mahmoud+Abd+El+Aleem+Ali+Ali), [Tarek El-Dabea](https://onlinelibrary.wiley.com/authored-by/El%E2%80%90Dabea/Tarek), [Rafat M. El-Khatib](https://onlinelibrary.wiley.com/authored-by/El%E2%80%90Khatib/Rafat+M.), [Aly Abdou](https://onlinelibrary.wiley.com/authored-by/Abdou/Aly), [Mohamed A. El Hamd](https://onlinelibrary.wiley.com/authored-by/El+Hamd/Mohamed+A.), [Ahmed M. Abu-Dief](https://onlinelibrary.wiley.com/authored-by/Abu%E2%80%90Dief/Ahmed+M.). Efficiency and development of guanidine chelate catalysts for rapid and green synthesis of 7-amino-4,5-dihydro-tetrazolo[1,5-*a*]pyrimidine-6-carbonitrile derivatives supported by density functional theory (DFT) studies. Appl. Organomet. Chem, [Volume37, Issue1](https://onlinelibrary.wiley.com/toc/10990739/2024/38/1)1, 2023, e7262

11. **What is about the future recommendations!**

Authors agree with the reviewer’s suggestion and justified additions are made in the manuscript.

12. **Names of all bacterial strains should write as italic.**

Done, wherever applicable.

13. **What about Molecular docking validation!**

Thank you for your valuable comments regarding the validation of molecular docking results. We appreciate the opportunity to clarify our methodology and point out the robustness of our study. In our study, it is noted that molecular docking was performed using AutoDock, which is a very popular and recognized tool in the structure-based drug discovery in terms of accuracy and sensitivity. To validate the docking results, we performed a detailed evaluation, including re-docking experiments, in which the reliability of the binding poses was confirmed. For example, the close alignment of the docking poses of the reference compound (TC) and derivative D_152 with the LasR active site, as indicated by nearly identical interaction profiles, underscores the validity of our docking predictions. In addition to the docking studies, molecular dynamics (MD) simulations were used to further validate the stability and binding interactions of compound D_152 with the LasR receptor. MD simulations were carried out over a 50 ns time span and indicated that compound D_152 constantly interacted with the LasR active site, thereby validating its role as a LasR antagonist. This approach provided dynamic insights into the dissolution of the protein's dimer, further confirming the functional relevance of D_152 as a quorum sensing inhibitor (QSI). We have included these details in the manuscript to ensure that the validation procedures and their significance are clearly communicated.

**References**

Sadiq S, Rana NF, Zahid MA, Zargaham MK, Tanweer T, Batool A, Naeem A, Nawaz A, Rizwan-Ur-Rehman, Muneer Z, Siddiqi AR. Virtual Screening of FDA-Approved Drugs against LasR of *Pseudomonas aeruginosa* for Antibiofilm Potential. Molecules. 2020 Aug 15;25(16):3723. doi: 10.3390/molecules25163723. PMID: 32824118; PMCID: PMC7466078.

Hevener KE, Zhao W, Ball DM, Babaoglu K, Qi J, White SW, Lee RE. Validation of molecular docking programs for virtual screening against dihydropteroate synthase. J Chem Inf Model. 2009 Feb;49(2):444-60. doi: 10.1021/ci800293n. PMID: 19434845; PMCID: PMC2788795.
